# Evolution of Dielectric Behavior of Regenerated Cellulose Film during Isothermal Dehydration Monitored in Real Time via Dielectric Spectroscopy

**DOI:** 10.3390/polym11111749

**Published:** 2019-10-24

**Authors:** Hao Zhao, Zhen Chen, Xianfeng Du

**Affiliations:** 1 Department of Applied Chemistry, School of Science, Anhui Agricultural University, Hefei 230036, China; 2 School of Tea and Food Science and Technology, Anhui Agricultural University, Hefei 230036, China; loveea@foxmail.com; 3 Beijing Advanced Innovation Center for Food Nutrition and Human Health, Beijing Technology and Business University, Beijing 100048, China

**Keywords:** regenerated cellulose film, dielectric spectroscopy, dehydration, dynamics, secondary relaxation

## Abstract

The dielectric relaxation behavior of a regenerated cellulose (RC) film during isothermal dehydration was monitored in real time via dielectric spectroscopy, in order to investigate on one hand the influence of water on its dynamics and the variation of microstructure and phase composition during dehydration on the other. The progression of water loss is clearly revealed by the evolution of the dielectric relaxation behavior with drying time, which suggests two distinctly different drying stages separated by a striking transition period. The dielectric relaxation behavior at the first drying stage is found overwhelmingly dominated by ionic motion, and that at the second stage is basically a result of molecular dynamics. The mechanisms of these relaxations are proposed, through which the influence of water on the dynamics of the RC film and the variation of the microstructure and phase composition of the film at different hydration state are discussed in detail. An interesting finding is that highly ordered but noncrystalline arrangement of cellulose molecules exists, but it can be formed only when the film is in specific hydration state. This study demonstrates that dielectric spectroscopy is an effective tool in real-time monitoring kinetic process.

## 1. Introduction

Regenerated cellulose (RC) attracts tremendous attention in recent decades, because it is advantageous over native cellulose in many aspects, such as greater diversity in structure, morphology, and functionality, while still holds the green nature [1,2,3]. Two categories of methods, chemical derivatization and physical dissolution, are generally used to prepare RC materials. Besides being much simpler, the latter is way more environmentally friendly than the former, thanks to less consuming of chemicals and no involvement of chemical reactions [2]. Physical dissolution method generally involves a dissolution process followed by a regeneration process. Depending on the regeneration process, various forms (fibers, beads, gels, etc.) of RC materials can be fabricated. Among these forms, RC film is an important one, which has long been used as packaging materials and has great potential in preparation of advanced composite materials [1,2,3,4,5]. 

A dehydration process is often needed to prepare anhydrous RC film, especially when water is the coagulant or residuals need to be removed through dialysis in water. Although the properties of RC film are primarily determined by the dissolution and coagulation process [2,3], novel microstructure and morphology may be achieved at the dehydration stage when special conditions like external force are applied [6,7,8,9]. The dehydration process of water-saturated RC film is a kinetic process of water loss, accompanied by evolution of phase composition and rearrangement of cellulose molecules. It is thus significant to monitor this process, in real time, to investigate how and under what condition the superstructure is formed. In addition, the influence of water on the properties of cellulose is always a topic of significance, because cellulose is highly hygroscopic and the influence is often dramatic. Therefore, real-time monitor on the dehydration process, in which a broad range of hydration state presents, could provide a fuller picture on the influence. 

Dielectric spectroscopy is a versatile method in the characterization of cellulosic materials, which inspects microscopic dynamics (either diffusional or orientational) of a material and is able to provide unique information regarding the structure and composition on molecular level [10,11,12,13,14,15]. Due to the non-invasion nature as well as other merits like rapidness in measurement, easy instrumentation, and simple sampling requirement [16,17], dielectric spectroscopy is also an effective tool in in-situ and real-time monitoring of kinetic processes, such as annealing of ultrastable glass [18], isothermal crystallization [19,20], and cell division [21]. 

The purpose of this study is to provide an insight into the interaction between water and cellulose under such a specific circumstance. To this end, the dielectric relaxation behavior of a RC film during an isothermal dehydration process was monitored in real time by means of dielectric spectroscopy. Through elaborate analysis on the characteristics of observed dielectric relaxations, the evolution pattern of these relaxations with loss of water was revealed, by which the influence of water on the dynamics of the film and the variation of the microstructure and phase composition under dehydration was discussed in detail. 

## 2. Materials and Methods

### 2.1. Materials

Cellulose powder was extracted from corn straw in light of the method proposed by Lu & Hsieh [22]. In brief: corn straw was thoroughly washed with deionized water and then dried in air for a week. The dry, cleaned corn straw was smashed and corn straw powder was collected from smashed straw through a 100-mesh screen. The powder was then treated with toluene/ethanol mixture to remove wax, pigments and oils. The de-waxed powder was then treated with acidified sodium chlorite and sodium hydroxide to dissolve lignin and to remove hemi-cellulose and silica, respectively. White cellulose powder thus obtained was finally frozen by liquid nitrogen and freeze-dried to remove excess water. The purity of cellulose powder was determined to be 91.63% with Technical Association of Pulp and Paper Industry (TAPPI) test method T203. Lithium Chloride (Purity > 99%), Silver Nitrate (Purity > 99%), and *N,N*-dimethylacetamide (DMAc, Anhydrous, 99.8%) were purchased from Sinopharm Chemical Reagent Co., Ltd. (Shanghai, China) and used without further purification.

### 2.2. Preparation of Cellulose Film

The cellulose powder was firstly activated in DMAc by stirring at 160 °C for 30 min. LiCl/DMAc (18 g/mL) solution was then added into the mixture, which was stirred at 110 °C for another 30 min. The mixture was subsequently cooled down to room temperature and stirred overnight, and transparent cellulose solution was obtained. All these steps were proceeded under anoxic environment. Transparent cellulose film was prepared by casting the cellulose solution on silicon wafer followed by drying in oven for 24 h at 45 °C. The as-obtained film was then soaked in deionized water for several days, and at meantime washed thoroughly from time to time with deionized water to remove LiCl and DMAc completely. The complete removal of the residues was confirmed by continuous titration of the supernatant with AgNO_3_ solution until no white precipitation was observed. After surface water being absorbed by fleece-free tissues, the transparent swollen film was cut in suitable shape and subject to dielectric measurement. The remaining film was weighed and then dried at 105 °C under vacuum until a constant weight was achieved. The moisture content (MC) of the film was determined by the weight loss of the sample thus dried, which is 49%. 

### 2.3. Real-Time Dielectric Monitoring

Dielectric measurements were carried out with a Novocontrol Broadband Dielectric Spectrometer (Concept 80, Novocontrol Technologies, Montabaur, Germany). To avoid direct blow by the nitrogen gas, a BDS 1308 cell was used, but the cell closing plate was removed so that the sample would not be vacuum sealed. The swollen cellulose film with a thickness of about 1 mm was sandwiched between the parallel electrodes and put into the chamber of the BDS 1308 cell, which was then mounted in the Novocontrol BDS 1200 sample holder for dielectric measurement. Considering that the film would be shrunk during the measurement, a spring was put in between of the upper electrode and the sample holder, so as to keep a solid contact with the sample throughout of the monitoring process. Dielectric spectrum with a frequency range of 0.05 Hz–10 MHz was recorded every half an hour (36 h in total) at a temperature of 20 °C. The temperature was controlled by the Quatro Cryosystem that is coupled to the spectrometer. 

### 2.4. Dielectric Spectra Analysis

The following fitting function containing a sum of several Havriliak-Negami (HN) functions and contribution from direct current (dc) conductivity ($\sigma_{\mathrm{DC}}$) was employed to quantitatively analyze the complex dielectric permittivity spectra:

(1) $$\varepsilon *(\omega ,T)=\varepsilon_{\infty }+\sum_{j}\frac{\Delta \varepsilon_{j}}{{\left[1+{\left(i\omega \tau_{j}\right)}^{\alpha_{j}}\right]}^{\gamma_{j}}}+\frac{\sigma_{\mathrm{DC}}}{i\omega \varepsilon_{0}}$$

where ε* is complex dielectric permittivity, which is functions of angular frequency (ω) and temperature (T), $\varepsilon_{\infty }$ is the dielectric constant in the high-frequency limit, Δε is the relaxation strength, τ is the characteristic relaxation time, α and γ quantify the symmetric and asymmetric broadening, respectively, j is the number of dielectric relaxations, and $\varepsilon_{0}$ is the permittivity of vacuum. 

It is always puzzling to perform accurate fit on entangled dielectric relaxation spectra with superposition of many relaxations. The logarithmic derivative (LD) method based on the following equation, however, is effective in resolving overlapping relaxation peaks due to peak sharpening [15,23,24,25].

(2) $$\varepsilon {ʺ}_{LD}(\omega )=-\frac{\pi }{2}\frac{\partial \varepsilon }{\partial \ln\omega }\approx \varepsilon {ʺ}_{Rel}(\omega )$$

where $\varepsilon {ʺ}_{LD}$ and $\varepsilon {ʺ}_{Rel}$ denote the derivative dielectric loss and the measured dielectric loss, respectively. To ensure high accuracy and reliability of the fits, simultaneous fitting on the curves of experimental dielectric loss (εʺ), dielectric constant ($\varepsilon^{ʹ}$), and derivative dielectric loss ($\varepsilon {ʺ}_{LD}$), coupled with nonlinear least-squares method, was employed to perform the fitting. The fitting procedure was described in detail in a previous study.

## 3. Results and Discussion

### 3.1. Evolution of the Dielectric Relaxation Behavior of RC Film during Isothermal Drying

**Figure 1:**
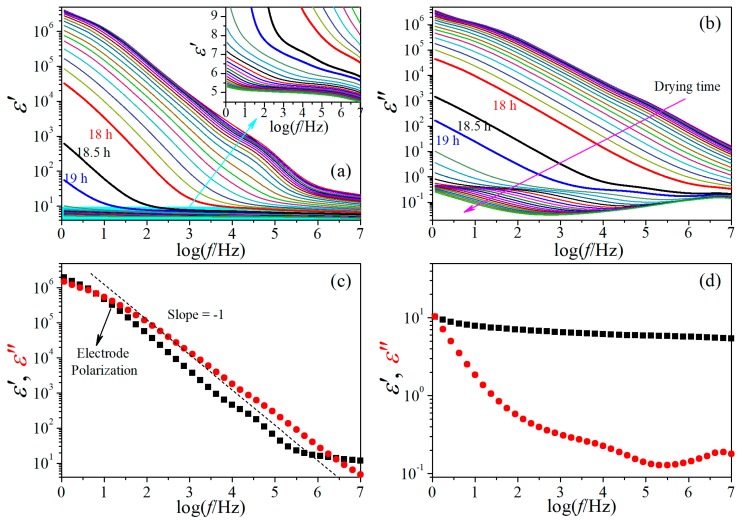
Variation of (**a**) dielectric constant and (**b**) dielectric loss of regenerated cellulose film with time during drying at 20 °C. The inset in (**a**) is the enlargement of dielectric constant spectra of the film after 19 h drying. Representative dielectric spectra at drying time of 10 h (**c**) and 22 h (**d**). The dashed line with a slope of −1 in (**c**) indicates dc conductivity.

Figure 1a,b shows the evolution of the dielectric behavior of the RC film with time during the isothermal drying process. A transition period around 18.5 h can be easily observed from both the $\varepsilon^{ʹ}$- and εʺ-spectra, which is characterized by remarkable decrease in magnitude when passing through the transition period. The whole drying process is thus divided into two distinctly different stages by the transition period. For simplicity, we call the drying stage at 0–18 h the first drying stage and that at 18.5–36 h the second drying stage hereinafter. 

A representative dielectric behavior of the RC film at the first drying stage is displayed in Figure 1c, from which one can notice that the value of εʺ surpasses that of $\varepsilon^{ʹ}$ already at frequencies around 10^6^ Hz. This suggests the dielectric behavior is overwhelmingly dominated by ionic motion. At frequencies lower than 100 Hz, obvious electrode polarization (EP) effect can be observed, which exhibits as a huge saddle-like peak in the εʺ-spectra. Though strongly influenced by EP, the relaxation at around 10^5^ Hz can be easily distinguished, and another one at its low frequency side (10^3^–10^4^ Hz) is still detectable. The dielectric behavior of the RC film at the second drying stage, on the other hand, is strikingly different, as shown in the representative case in Figure 1d. In the whole investigation frequency range, the value of εʺ is smaller than that of $\varepsilon^{ʹ}$, indicating the dielectric behavior is dominated by molecular motions. Due to distinct difference between these two stages, the evolution of the dielectric relaxation behavior with drying time is discussed individually as follows. 

#### 3.1.1. Dielectric Behavior at the First Drying Stage (0–18 h)

**Figure 2:**
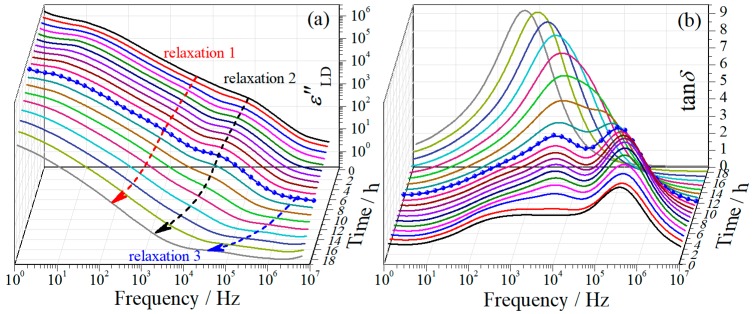
Frequency dependences of (**a**) derivative dielectric loss $\varepsilon {ʺ}_{LD}$ and (**b**) loss tangent tanδ of regenerated cellulose film at different drying moments at the first drying stage. The arrows in (a) are used only for guiding eyes. The blue symbol-line curve indicates a transition moment.

Due to the strong influence from EP effect, the dielectric relaxations at the first drying stage are difficult to be discriminated from the $\varepsilon^{ʹ}$- and εʺ-spectra. Other representations, namely derivative dielectric loss ($\varepsilon {ʺ}_{LD}$) and loss tangent ($\tan\delta =\varepsilon ʺ/\varepsilon^{ʹ}$), were then employed to demonstrate the evolution of the dielectric behavior, which are displayed in Figure 2a,b, respectively. From the $\varepsilon {ʺ}_{LD}$-spectra, one can notice that the relaxation initially locating at around 10^5^ Hz (relaxation 2 hereinafter) is more prominent and the one (relaxation 1 hereinafter) at its lower frequency side (10^3^–10^4^ Hz) becomes visible, as compared with the εʺ-spectra (see Figure 1b). These two relaxations are even more distinguishable in the tanδ-spectra, as shown in Figure 2b where two well-separated peaks can be evidently seen. Both representations indicate a transition moment in the vicinity of 10 h (the blue symbol-line curve): Before the transition moment, both relaxations shift towards lower frequency range with drying time, with slight variation in magnitude; after the moment, however, obvious decrease in magnitude of both relaxations can be observed in the $\varepsilon {ʺ}_{LD}$-spectra and a merge of these two relaxations is observed in the tanδ-spectra. Meanwhile the shifting of both relaxations towards lower frequency range is accelerated after the moment. In addition to these two large relaxations, a small relaxation (relaxation 3 hereinafter) is observed in their high frequency side, which becomes appreciable after about 10 h drying. 

**Figure 3:**
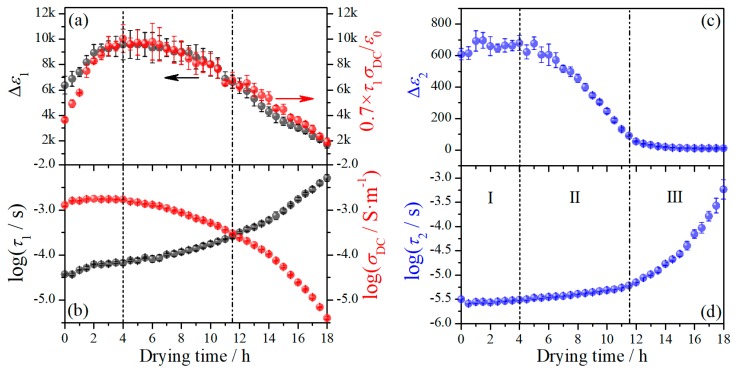
(**a**) Variation of the relaxation strength of relaxation 1 and the product of dc conductivity and relaxation time with drying time; (**b**) variation of relaxation time of relaxation 1 and dc conductivity with drying time; variation of (**c**) relaxation strength and (**d**) relaxation time of relaxation 2 with drying time. The short dash and dash-dot-dot lines indicate the transition moments.

To investigate the mechanisms of these relaxations, the dielectric spectra were analyzed in line with Equation (1) using the fitting strategy mentioned above. While the tanδ-spectra cannot be directly fit with Equation (1), they were used to testify the reliability of the fits, through comparing to the tanδ curves converted from the final fitting curves. Relaxation characteristics including relaxation strength (Δε) and characteristic relaxation time (τ) were determined in this way, the former denoting the magnitude and the latter reflecting the dynamic feature of the relaxations. The variation of Δε and τ of relaxation 1 and 2 with drying time at the first drying stage is demonstrated in Figure 3. $\Delta \varepsilon_{1}$ and $\Delta \varepsilon_{2}$ are initially large, which are of the order of thousands and hundreds dielectric units, respectively. The values of symmetric broadening parameter *α* (see Equation (1)) are around 0.9 and 1 for relaxation 1 and relaxation 2, respectively, suggesting both relaxations are of Debye type. The relaxation 3 has a relatively fairly small relaxation strength with a broad relaxation time distribution (*α* is around 0.6), indicative of an origin of molecular motion. This relaxation thus should be the $\beta_{\mathrm{wet}}$- relaxation and will be discussed in Section 3.1.2.

A transition moment at 11.5 h can be noticed from Figure 3, which is consistent with the observation from Figure 2; in addition, another transition moment at about 4 h can also be observed, which is more evidently revealed in the variation of Δε with drying time. These two transition moments thus divide the first drying stage into three sub-stages, as pointed out in Figure 3. At the sub-stage I, both $\Delta \varepsilon_{1}$ and $\tau_{1}$ have an obvious increase with drying time while $\Delta \varepsilon_{2}$ and $\tau_{2}$ keep nearly constant. At the sub-stage II, while both $\Delta \varepsilon_{1}$ and $\Delta \varepsilon_{2}$ decrease with drying time, the latter has a much stronger dependence on time. Both increasing with drying time, $\tau_{1}$ has a notably stronger dependence on drying time than that of $\tau_{2}$. At the sub-stage III, the decrease of $\Delta \varepsilon_{1}$ with drying time seems following the same tendency as at the sub-stage II, but the dependence of $\Delta \varepsilon_{2}$ on drying time suddenly becomes fairly weak. The increase of $\tau_{1}$ and $\tau_{2}$ with drying time is apparently accelerated at this sub-stage, with the change of the latter being much more pronounced. The variation of dc conductivity ($\sigma_{\mathrm{DC}}$) with drying time is also presented in Figure 3b, from which one can see $\sigma_{\mathrm{DC}}$ keeps nearly constant at the sub-stage I, followed by a decrease at the sub-stage II and a more pronounced decrease at the sub-stage III. The variation of $\sigma_{\mathrm{DC}}$ with drying time is overall inverse of that of $\tau_{1}$ and $\tau_{2}$, indicative of a strong coupling of them. This hence implies that both relaxation 1 and 2 could have the same or fundamentally similar mechanism to that of dc conductivity.

With a MC as high as 49%, the RC film is in a jelly state and composed of regenerated cellulose molecules and water. Since obvious degradation is not expected when cellulose is regenerated from LiCl/DMAc solution [2,26], the cellulose molecules should hold its native molecular structure but could be mostly rearranged forming new morphology or ordering. The film in its initial state thus can be considered as a network of rearranged cellulose molecules immersing in water, where water is the continuous phase consisting of free water and bound water. The bound water could present in three states, including freezable loosely bound water, non-freezable loosely bound water, and tightly bound water, according to our previous study [15]. On the other hand, the film has been fully soaked in and washed with water, ionic species rather than H^+^ ions should be negligible. Accordingly, the ionic conduction in the film could be mainly ascribed to diffusive migration of H^+^ ions in the water phase and charge transport via hopping of H^+^ ions amongst hydroxyl groups on the cellulose molecules in the polymer phase. The hop of H^+^ ions is believed to be analogous to the hopping conduction in disordered solids like ionic conductive glasses, which is described by the random free-energy barrier model [27]. According to the model, conductivity is ascribed to the hop of charge carriers in spatially randomly varying energy barriers. It is constant at low frequencies as a result of charge transport on infinite “percolation” paths (dc conductivity) and becomes frequency dependent at higher frequency range where charge transports via hopping in finite clusters (ac conductivity), which behaves as a dielectric relaxation. Due to the same underline mechanism, the dc conductivity is correlated with the strength of the relaxation of ac conductivity by the empirical Barton-Nakajima-Namikawa (BNN) relation expressed as [27,28,29],

(3) $$\sigma_{\mathrm{DC}}=p\varepsilon_{0}\Delta \varepsilon /\tau_{m}$$

where p is correlation factor which varies with the microscopic structure and composition of the system, and $\tau_{m}$ is the characteristic relaxation time, which approximates the hopping time defined as the time for charge carriers attempting to overcome the highest energy barrier [27,30,31]. In Figure 3a, the product of $\tau_{1}$ and $\sigma_{\mathrm{DC}}$ with a correlation factor value of 0.7 is displayed. Interestingly, the variation of this product is nearly identical to that of $\Delta \varepsilon_{1}$ at all three sub-stages, which suggests that the relaxation 1 have the same underline mechanism as dc conductivity.

On the other hand, the BNN relation was found not applicable to the case of relaxation 2, implying that this relaxation arises from a different relaxation mechanism. Judging from the facts that this relaxation is also coupled with dc conductivity and it is Debye type, we consider this relaxation ascribed to interfacial polarization, namely the Maxwell-Wagner effect [32,33]. The relaxation characteristic of this relaxation, accordingly, is dependent not only on ionic conduction but also on the permittivity of involved phases, the phase composition, and the local properties at the interfaces.

#### 3.1.2. Dielectric Behavior at the Second Drying Stage (18.5–36 h)

**Figure 4:**
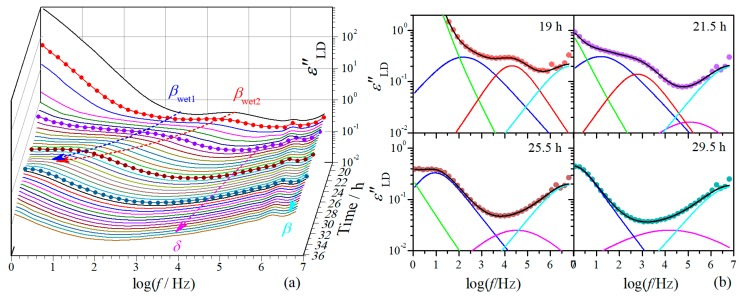
(**a**) Frequency dependence of derivative dielectric loss $\varepsilon {ʺ}_{LD}$ of regenerated cellulose film at different drying moments at the second drying stage. The symbol-line curves are randomly selected moments used as fitting examples and the arrows are for guiding the eyes. (**b**) Fits on the derivative dielectric loss spectrum at randomly selected moments as highlighted in (a). The lines are fitting curves, black: total fit, green: ionic motion relaxation, blue: $\beta_{\text{wet}1}$-relaxation, red: $\beta_{\text{wet}2}$-relaxation, magenta: *δ*-relaxation, cyan: *β*-relaxation.

While the dielectric relaxation behavior is dominated by ionic motion at the first drying stage, it is dominated by the molecular dynamics at the second stage. The evolution of dielectric relaxation with drying time at this stage is demonstrated in Figure 4a, where it can be noticed that the magnitude of the relaxations is strikingly smaller than that at the first drying stage. The dielectric relaxation behaviors at four randomly selected drying moments are demonstrated in Figure 4b, which look similar to those of native cellulose with different MC at 20 °C [15]. Typical β-relaxation is observed in the frequency range of 10^6^–10^7^ Hz, which is consistent with that found in native celluloses of different origins [11,15,34]. The β-relaxation seems barely changed with drying time. At the low frequency side of this relaxation, δ-relaxation is revealed after 20 h, before which it should be completely covered by its neighboring relaxations. While only one outstanding $\beta_{\mathrm{wet}}$-relaxation can be observed in native cellulose, it is very interesting that, from 18.5 h two well-separated relaxations appear in the frequency range where typical $\beta_{\mathrm{wet}}$-relaxation occurs. More interestingly, these two relaxations are approaching with each other with drying time and eventually merge as a single relaxation at around 27 h.

**Figure 5:**
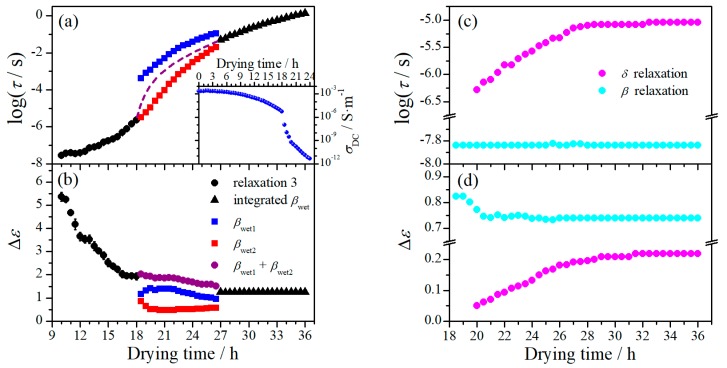
(**a**) Variation of the characteristic relaxation time of $\beta_{\text{wet}}$-relaxation and dc conductivity (inset) with drying time; (**b**) variation of relaxation strength of $\beta_{\text{wet}}$-relaxation with drying time; variation of (**c**) characteristic relaxation time and (**d**) relaxation strength of *δ*- and *β*-relaxation with drying time. The short dashed line is for guiding the eyes.

To give a closer look at the variation of these relaxations, the dielectric spectra were analyzed and their Δε and τ are plotted against drying time in Figure 5. For the β-relaxation, it is noteworthy that both Δε and τ are barely changed during the second drying stage. The slight variation of Δε in the period of 18.5–20 h should be due to incorporation of the *δ*-relaxation. This indicates that β-relaxation is hardly influenced by the loss of water at this drying stage. The δ-relaxation shifts smoothly towards lower frequency range with drying time at the period of 20–27 h, characterized by nearly linear increase of Δε and logτ. The dependence on drying time of this relaxation becomes extremely weak after 27 h.

The unusual behavior of $\beta_{\mathrm{wet}}$-relaxation is more clearly demonstrated in Figure 5a,b. This relaxation shows up at the first drying stage as a single relaxation (relaxation 3 in Figure 2), shifting towards low frequencies with decreasing Δε with drying time. After the transition period around 18.5 h, two well-separated relaxations suddenly appear. The higher-frequency relaxation ($\beta_{\mathrm{wet}2}$ in Figure 4 and Figure 5) seems still following the variation tendency in τ as that of the relaxation 3, but its Δε is remarkably decreased (by more than a half). The lower-frequency relaxation ($\beta_{\mathrm{wet}1}$ in Figure 4 and Figure 5), on the other hand, appears at frequencies about two orders lower than the higher-frequency one, and its Δε is also much smaller than that of the relaxation 3. These two relaxations keep approaching with each other during the period of 18.5–27 h and merge into an integrated relaxation at 27.5 h. Since the magnitude of the relaxation 1 and 2 are large, $\beta_{\mathrm{wet}1}$-relaxation could have already existed at the first drying stage but be totally covered by the ionic motion relaxations. In other words, this relaxation may always exist but can only be resolved after the transition period. However, we prefer to believe both $\beta_{\mathrm{wet}1}$- and $\beta_{\mathrm{wet}2}$-relaxations are new relaxations that are originated from the relaxation 3 and separate at certain transition moment. We judge this based on the following facts: (1) Both relaxations have quite similar relaxation characteristics to the relaxation 3, with fairly broad relaxation time distribution and small relaxation strength. They should have a similar molecular mechanism to the relaxation 3. (2) As far as τ is concerned, although the $\beta_{\mathrm{wet}2}$-relaxation seems to have an ideal variation path bridging the relaxation 3 and the integrated $\beta_{\mathrm{wet}}$-relaxation, it is expected that there should be an abrupt increase in τ after the transition period, which could follow a variation tendency as suggested by the dashed line shown in Figure 5a. Note that a cliff-like drop in dc conductivity is observed after the transition period (more than three orders decrease within 2 h), as shown in the inset of Figure 5a, which indicates a typical percolation phenomenon and suggests a critical change in the phase composition. Accordingly, it is plausible to consider that $\beta_{\mathrm{wet}1}$- and $\beta_{\mathrm{wet}2}$-relaxation shifts inversely when separated from the relaxation 3, towards lower and higher frequency range, respectively. (3) As far as Δε is concerned, we found the summation of Δε of these two relaxations follows perfectly with the variation tendency of the relaxation 3 and bridges well to the integrated $\beta_{\mathrm{wet}}$-relaxation, as can be seen in Figure 5b. It should be noted here that Δ*ε* of $\beta_{\mathrm{wet}}$-relaxation of cellulose has very weak dependence on MC [15]. Accordingly, we suggest that the $\beta_{\mathrm{wet}}$-relaxation is initially an integrated relaxation but evolves into two separated sub-relaxations after the transition period, which eventually merge into an integrated one at a succeeding transition moment.

**Figure 6:**
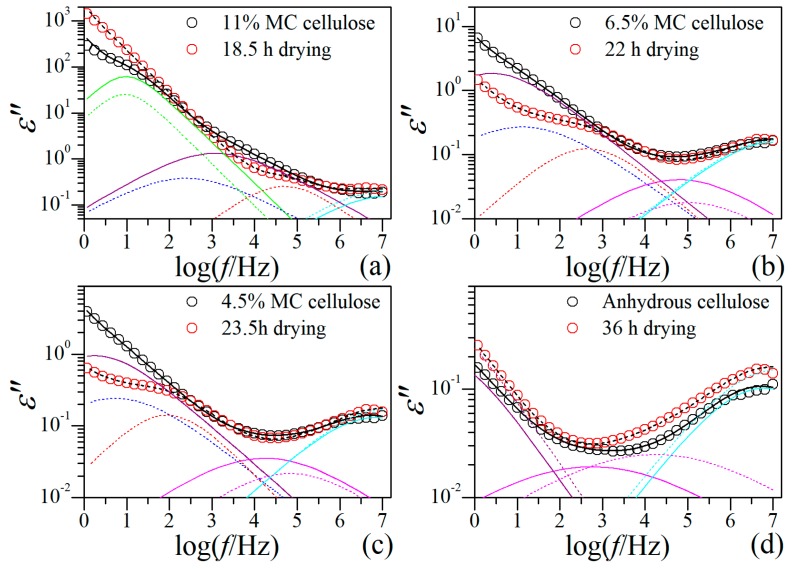
Comparison on the dielectric relaxation behavior between native cellulose with different moisture content and regenerated cellulose film at different drying moments.

A question arises here, whether the unusual behavior of $\beta_{\mathrm{wet}}$-relaxation is originated from the drying process or from the structural nature of the RC film? Since the morphology and local structure of the film and the arrangement of cellulose molecules therein have been formed during the coagulation process, we believe they could not be significantly changed when the film was soaked in water, but the cellulose network could be considerably expanded due to swelling by water. The isothermal drying process thus should be mainly a process of water loss accompanied by shrinkage of cellulose network. We therefore consider that the unusual behavior is a result of the structural nature of the film instead of the drying process. To clarify this point, the dielectric behaviors at 20 °C of native cellulose with different MC are compared with those of RC film at certain drying moments. The native cellulose samples that were previously studied are the same cellulose sample used for the RC film preparation in this study [15]. The drying moments are selected on the basis of the best match in relaxation behavior at high frequency range between the RC film and native cellulose, considering that *β*- and *δ*-relaxation are barely influenced by the drying process. The comparisons are demonstrated in Figure 6, from which one can notice that the native cellulose with higher MC is always comparable to the RC film at smaller drying moment, suggesting water loss is the primary factor inducing the evolution of the dielectric relaxation behavior. It is very impressive that, the two sub$\beta_{\mathrm{wet}}$-relaxations in the RC film always locate within the single big $\beta_{\mathrm{wet}}$-relaxation observed in native cellulose, while other types of relaxations are basically consistent. This result is a further confirmation of our previous suggestion that $\beta_{\mathrm{wet}1}$- and $\beta_{\mathrm{wet}2}$-relaxations are assigned to $\beta_{\mathrm{wet}}$-relaxation. The result also strongly advises that the unusual relaxation behavior of $\beta_{\mathrm{wet}}$-relaxation in the RC film is a consequence of the unique local structure or arrangement of regenerated cellulose molecules rather than the drying process (the moisture content).

The regeneration of cellulose film from LiCl/DMAc solution is a complex process involving a cellulose dissolution process and a coagulation process [3,35,36,37]. A large number of factors can affect this process [7,37], such as the nature and activation state of native cellulose, the coagulation solvent and condition, the external force applied during film-forming process, and of course temperature and pressure. As a result, the RC film can present great diversity in structure and morphology [6,7,8,38,39,40,41,42]. A concept of “nematic ordered cellulose” (NOC) was recently proposed by Kondo et al. [8,9,38], based on the discovery of a noncrystalline but highly ordered phase in the RC film prepared from LiCl/DMAc solution [7]. The formation of ordered supermolecular structure of RC film was aware even earlier [6], which was pointed out to be a general phenomenon. In the current case, the RC film was prepared by slow precipitation, and highly ordered yet noncrystalline microstructure could be formed, which holds the most probable cause of this unusual behavior. Following the NOC concept [9,38], the highly ordered phase has a layered structure, and in each layer the cellulose chains are fully stretched and aligned preferentially to certain direction. This formation suggests two types of cellulose-water complex, namely the one within the layers and the one between the layers. Since $\beta_{\mathrm{wet}}$-relaxation of cellulose is believed to ascribed to the dynamics of complex composed of cellulose molecule and tightly bound water molecules [15], it is justifiable that two sub -$\beta_{\mathrm{wet}}$-relaxations appear in current case, considering that the dynamics of the two types of cellulose-water complex should be different. Since the cellulose-water complex within the layer is much more restricted as compared with that between the layers, its dynamics should be responsible for the $\beta_{\mathrm{wet}1}$-relaxation, and the $\beta_{\mathrm{wet}2}$-relaxation should arise from the dynamics of complex between the layers.

### 3.2. Progression of Water Loss and Its Influence on the Dielectric Relaxation Behavior of RC Film

Although the dielectric behavior of the RC film has remarkably different characteristics at the two drying stages, overall a successive variation with drying time is identified, and the direct factor inducing this variation is undoubtedly the loss of water. As previously pointed out, the RC film contained considerable amount of free water in addition to bound water. Obviously, free water would be lost first during the drying process, followed by the loss of bound water in a sequence of freezable, non-freezable, and tightly bound water. The loss of water definitely triggers change of the film in phase composition and local structure, and therefore influences its dielectric behavior. As well, the evolution of the dielectric behavior, in turn, reflects the variation of hydration state and microstructure of the film.

The impressive percolation phenomenon exhibited in dc conductivity as shown in Figure 5a indicates a crucial change in the phase composition of the RC film at the transition period. The crucial change should be due to a transition of water phase from continuous phase to dispersed phase, because the dielectric relaxation behavior is dominated by molecular motions after the transition. Since the dielectric behavior of the RC film with 18.5 h drying is consistent with that of the native cellulose with 11% MC, in which considerable amount of freezable loosely bound water still exists [15], the water that is lost at the first drying stage should be free and freezable loosely bound water. Three sub-stages are distinguished by two transition moments at 4 h and 11.5 h at this drying stage. According to the evolution of the dielectric relaxation behavior, we judge that the sub-stage I is a process of pure loss of free water, the sub-stage II is a loss of both free water and freezable loosely bound water, and the sub-stage III is a loss of freezable loosely bound water, and a phase transition occurs at the transition moment around 11.5 h.

As discussed above, the relaxation 1 has the same underline mechanism as dc conductivity, while the relaxation 2 should be due to interfacial polarization. According to the Einstein and Einstein-Smoluchowski relations, dc conductivity is related to the hopping time ($\tau_{e}$) by [30,31]

(4) $$\sigma_{\mathrm{DC}}=\frac{nq^{2}\lambda^{2}}{2k_{B}T\tau_{e}}$$

where n is the effective number density of the charge carrier, q is the elementary electric charge, $k_{B}$ is the Boltzmann constant, T is absolute temperature, and *λ* is the hopping length. Consider $\tau_{e}\approx \tau_{1}$, $\Delta \varepsilon_{1}$ (on the order of product of $\sigma_{\text{DC}}$ and $\tau_{e}$) is mainly functions of *n* and *λ*, according to Equation (4). At the sub-stage I, free water is the dominant phase and cellulose molecules is fully outstretched. Loss of free water would not significantly change the local structure of cellulose molecules (still fully hydrated), hence *λ*, which is the order of distance between two adjacent hydroxyl groups on cellulose molecule in the current case, keeps barely changed. However, the loss of water leads to an increase in n, and meanwhile an increase in $\tau_{e}$ considering that a more crowded charges may induce a larger energy barrier and thus a longer hopping time. Consequently, dc conductivity has a weak variation due to proportionally concurrent change of n and $\tau_{e}$, yet $\Delta \varepsilon_{1}$ noticeably increases as a result of increasing n. On the other hand, the situation at the interface between water and cellulose should not be considerably changed, as long as free water still exists and hence the electric properties of whole water phase do not obviously change. This is manifested by the dielectric behavior of the relaxation 2 at this sub-stage, which has negligible dependence on time.

After 4 h drying, further loss of free water and subsequent loss of freezable loosely bound water would induce significant change in phase composition and local structure of cellulose network. The network would be squeezed due to shrinkage of the film, giving rise to denser molecular arrangement. Decreasing λ with drying time could be expected as a consequence, together with increasing $\tau_{e}$ due to gradually enhanced steric hindrance as well as stronger association between H^+^ ion and hydroxyl group. The change of n could be slight, because while the density of charge carrier would increase with water loss the percentage of charge carrier able to achieve effective hop could be decreased. This should be a common feature in structural change at both sub-stage II and sub-stage III, where the variations of dc conductivity and relaxation 1 are always consistent with each other thanks to the same dependence on the above quantities. In addition to the structural change, the phase composition of the film also varies with loss of water. The transition moment at around 11.5 h signifies such a critical change in phase composition, after which we believe water is no longer the dominant phase and a bicontinuous phase composition is formed. Besides, free water should have been totally lost at this moment. As a result, the variation of $\sigma_{\mathrm{DC}}$ and $\tau_{1}$ with time at the sub-stage III becomes more pronounced, note that cellulose molecules are much more rigid since plasticizing effect from water is substantially reduced at this sub-stage. The evolution of the relaxation 2 is a result of Maxwell-Wagner effect subject to the change of phase composition. Simply speaking, the Maxwell-Wagner effect results from difference in electric properties of two adjacent phases, the magnitude of the relaxation therefrom depends on the magnitude of the difference and volume fraction of the constituent phases, and the relaxation time is determined by the rate of charge building-up at the interface. As compared with free water, bound water has obviously smaller $\varepsilon^{ʹ}$ and σ, which decrease exponentially as approaching to cellulose phase. Accordingly, $\Delta \varepsilon_{2}$ decreases intensively at the sub-stage II due to decreasing difference in electric property and volume fraction of water. At the sub-stage III, since both electric property and volume fraction of water are comparable to the cellulose phase, $\Delta \varepsilon_{2}$ is small and slightly changes with drying time. The variation of $\tau_{2}$ is similar to that of $\tau_{1}$, because it is mainly determined by the ionic conduction in the film.

The most striking change occurs at the transition period around 18.5 h, which definitely signifies another critical change in phase composition, after which cellulose becomes the dominant (continuous) phase while water exists only within the hydration layer. The dielectric relaxation behavior at the second drying stage is thus attributed to molecular motions subject to the influence from bound water. The unusual behavior of the $\beta_{\mathrm{wet}}$-relaxation and the variation of the δ-relaxation suggest a transition moment at about 27 h, as can be seen in Figure 5. According to our previous study [15], while both tightly bound water and non-freezable loosely bound water are directly involved in the δ- relaxation, only the latter influences its dynamical performance. Both Δε and τ of this relaxation increase with drying time before the transition moment and keep nearly constant afterwards. We therefore believe this transition moment corresponds to total loss of loosely bound water. The appearance of two sub$\beta_{\mathrm{wet}}$-relaxations seems to suggest that ordered arrangement of cellulose molecules can only be formed when most freezable loosely bound water disappears. Before that, cellulose molecules may possess high degree of freedom due to existence of sufficient amount of water, so that highly ordered alignment could hardly be achieved. A very faint transition at around 21 h can be identified from the variation of Δε of the two sub-relaxations and from that of $\sigma_{\text{DC}}$ (Figure 6a,b), which might be a moment of total loss of freezable loosely bound water. The approaching of $\beta_{\mathrm{wet}1}$- and $\beta_{\mathrm{wet}2}$-relaxation with drying, in our opinion, is a result of densification of the film induced by continuous loss of bound water. With loss of bound water, the layered structure of cellulose molecules becomes more and more compact both horizontally and vertically, leading to more restricted dynamics of cellulose-water complex and hence longer relaxation time. However, the vertical change is more pronounced, because the surfaces of the layer are much more accessible to water molecules than its interior. Accordingly, the $\beta_{\mathrm{wet}2}$-relaxation, which arises from the dynamics of cellulose-water complex formed between the layers, dynamically has stronger dependence on the loss of water than the $\beta_{\mathrm{wet}1}$-relaxation. This is what can be observed in Figure 6a: while both sub-relaxations are shifting to lower frequency range, $\beta_{\mathrm{wet}2}$-relaxation shifts faster, which results in the eventual merge of the two sub-relaxations at around 27 h. After this moment, only tightly bound water exists, which is directly bound with the hydroxyl group (the first hydration layer). Therefore, the dynamics of cellulose-water complex is somehow uniform throughout of the film, giving rise to an integrated $\beta_{\mathrm{wet}}$-relaxation.

## 4. Conclusions

By real-time monitoring an isothermal dehydration process of cellulose film regenerated by physical dissolution via dielectric spectroscopy, the evolution of the dielectric relaxation behavior of the film with drying time was studied. Two distinctly different drying stages separated by a striking transition period around 18.5 h are identified, which corresponds to a phase transition of the film from water-dominant to cellulose-dominant.

At the first drying stage, two relaxations are observed, which are attributed to hop of hydrogen ion among hydroxyl groups on cellulose molecule and interfacial polarization respectively. The variation of these relaxations with drying time suggests two transition moments at 4 h and 11.5 h, which correspond to a pure loss of free water and a phase transition from water-dominant to bicontinuous phase composition.

At the second drying stage, the relaxation behavior of the film is basically dominated by molecular motion. Typical secondary dynamics of native cellulose including β-relaxation and δ-relaxation are observed, which locates at similar frequency range to those found in native cellulose and exhibits relatively weak dependence on drying time. The $\beta_{\mathrm{wet}}$-relaxation in the film, however, has an unusual behavior characterized by a sudden appearance of two sub-relaxations and a merge into one integrated relaxation at a transition moment around 27 h. This result suggests the existence of a highly ordered yet noncrystalline arrangement in the film. It also suggests that such an ordering can only be formed when freezable loosely bound water is totally lost.
